# Therapy development for spinal muscular atrophy: perspectives for muscular dystrophies and neurodegenerative disorders

**DOI:** 10.1186/s42466-021-00162-9

**Published:** 2022-01-04

**Authors:** Sibylle Jablonka, Luisa Hennlein, Michael Sendtner

**Affiliations:** Institute of Clinical Neurobiology, University Hospital of Wuerzburg, Versbacher Str. 5, 97078 Wuerzburg, Germany

**Keywords:** Motoneuron disease, Neurodegenerative disease, Muscular disease, Spinal muscular atrophy, Amyotrophic lateral sclerosis, Muscular dystrophy, Alzheimer disease, Parkinson disease, Clinical trial, Gene therapy

## Abstract

**Background:**

Major efforts have been made in the last decade to develop and improve therapies for proximal spinal muscular atrophy (SMA). The introduction of Nusinersen/Spinraza™ as an antisense oligonucleotide therapy, Onasemnogene abeparvovec/Zolgensma™ as an AAV9-based gene therapy and Risdiplam/Evrysdi™ as a small molecule modifier of pre-mRNA splicing have set new standards for interference with neurodegeneration.

**Main body:**

Therapies for SMA are designed to interfere with the cellular basis of the disease by modifying pre-mRNA splicing and enhancing expression of the Survival Motor Neuron (SMN) protein, which is only expressed at low levels in this disorder. The corresponding strategies also can be applied to other disease mechanisms caused by loss of function or toxic gain of function mutations. The development of therapies for SMA was based on the use of cell culture systems and mouse models, as well as innovative clinical trials that included readouts that had originally been introduced and optimized in preclinical studies. This is summarized in the first part of this review. The second part discusses current developments and perspectives for amyotrophic lateral sclerosis, muscular dystrophies, Parkinson's and Alzheimer's disease, as well as the obstacles that need to be overcome to introduce RNA-based therapies and gene therapies for these disorders.

**Conclusion:**

RNA-based therapies offer chances for therapy development of complex neurodegenerative disorders such as amyotrophic lateral sclerosis, muscular dystrophies, Parkinson’s and Alzheimer’s disease. The experiences made with these new drugs for SMA, and also the experiences in AAV gene therapies could help to broaden the spectrum of current approaches to interfere with pathophysiological mechanisms in neurodegeneration.

## Background

Spinal muscular atrophy (SMA) is the most common form of a lethal pediatric neuromuscular disorder with autosomal recessive inheritance. It is caused by homozygous loss of function (LOF) mutations of the *Survival Motor Neuron 1 (SMN1)* gene [170] on human chromosome 5(5q13.2). Thus, therapeutic approaches so far have focused on restoration of SMN expression. The specific architecture on human chromosome 5 with a second *SMN* gene (*SMN2*) is responsible for the cellular production of low levels of SMN protein that are not sufficient to maintain structure and function of motoneurons. *SMN2* differs from *SMN1* by a single C to T transition in exon 7, leading to increased skipping of exon 7 [180, 206]. Thus, approaches to suppress alternative splicing of this exon and an AAV9-based gene therapy for enhanced expression of the SMN protein in motoneurons have led to success in treating degeneration of motoneurons in this disease.

Restoration of protein expression is also a central goal for therapy development in Duchenne-and Becker-type muscular dystrophies [52, 72, 155]. Thus, oligonucleotide-based therapies as well as gene therapies are currently tested in these disorders. Experience with such therapies is rapidly progressing, and this also has impact on therapy development for other neurodegenerative disorders such as amyotrophic lateral sclerosis (ALS). Oligonucleotide therapies do not exclusively offer the chance to increase expression of proteins such as SMN, but also to repress expression of mutant proteins with pathological function in other neurodegenerative disorders. This offers further technical opportunities for interference with other neurodegenerative mechanisms.

Our review summarizes the development of antisense-oligonucleotide (ASO) and gene therapy for SMA, based on the literature search via PubMed.gov and released data from https://clinicaltrials.gov. The second part addresses opportunities and challenges associated with further development of these approaches for treatment of other neurodegenerative disorders and muscular dystrophies.

## Spinal muscular atrophy (SMA): disease mechanisms and identification of targets for therapy

### Disease presentation and classification of spinal muscular atrophy (SMA)

The severe form of proximal spinal muscular atrophy, also called Werdnig-Hoffmann disease [122, 123, 316], is the most common monogenetic lethal pediatric neuromuscular disorder. A milder form of proximal spinal muscular atrophy also exists that originally has been considered as a distinct neurological disease [160]. However, after the identification of the underlying gene defect [170], it became apparent that both diseases are caused by homozygous deletion of the *Survival Motor Neuron 1* (*SMN1*) gene on human chromosome 5q13.2. All forms of 5q-SMA (type 1–4) have an incidence of 1/6000–10,000 world-wide [77, 229, 231, 305]. SMA follows autosomal recessive inheritance. Dysfunction and loss of spinal motoneurons is the most prominent pathological feature causing weakness and atrophy, notably in proximal muscle groups, and respiratory failure.

Depending on disease onset and severity, SMA is classified into four types ranging from the most severe type 1 to intermediate type 2 and milder types 3 and 4 (with adult onset) [69, 70, 78, 230]. This classification mainly focuses on achieved motor milestones with the disadvantage of frequent overlap between different types. Thus, an additional classification has been introduced to cover dynamic changes in the clinical phenotype after therapy as well. This new classification distinguishes non-sitters (type 1–2), sitters (type 2–3) and walkers (type 3–4) [197], summarized in Table 1.

**Table 1:**
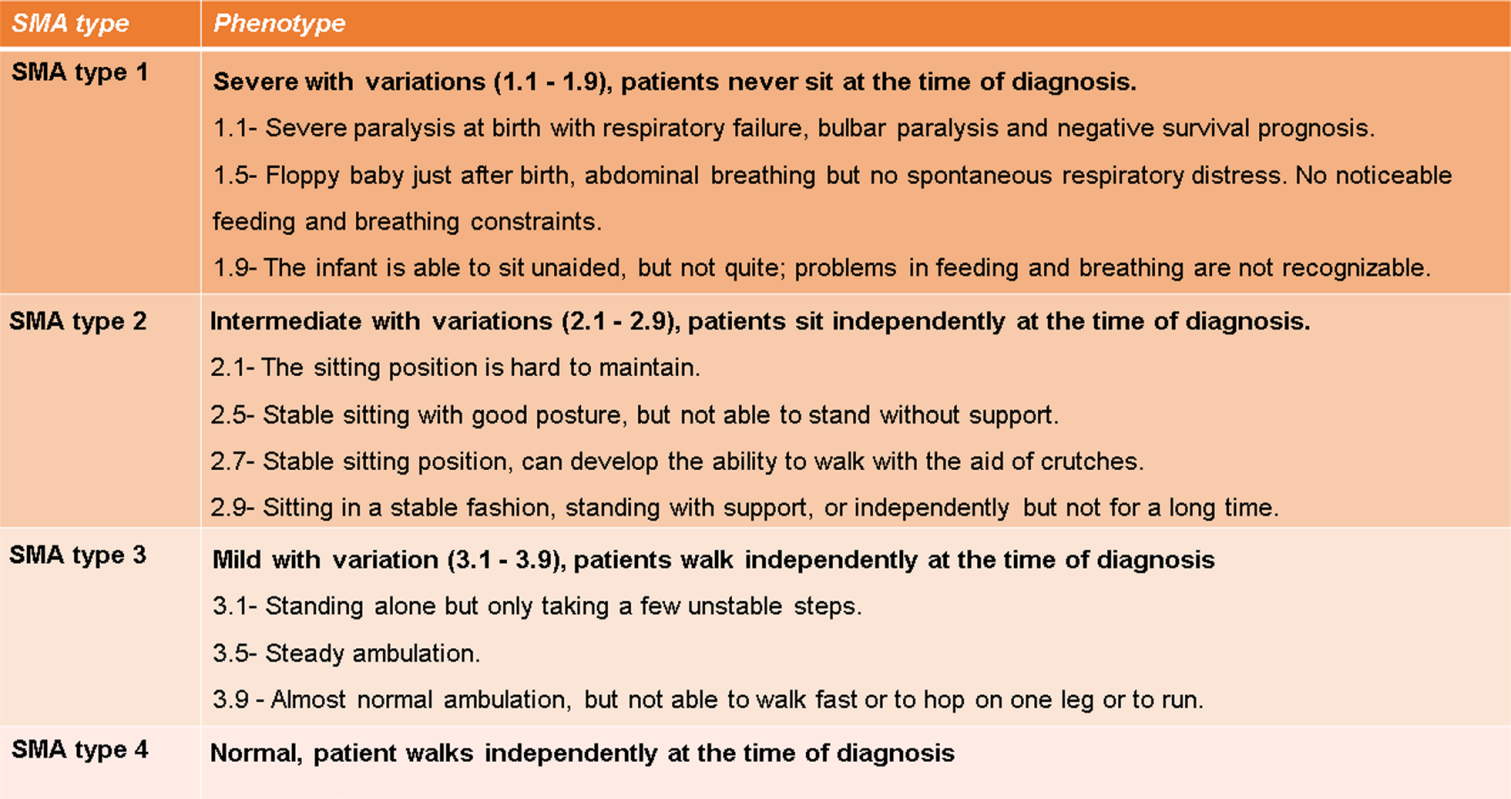
New classification of disease severity in SMA patients according to [69, 78, 230] and adapted to [70]

### SMA genetics

#### The two *survival motor neuron* genes: *SMN1* and *SMN2*

**Fig. 1:**
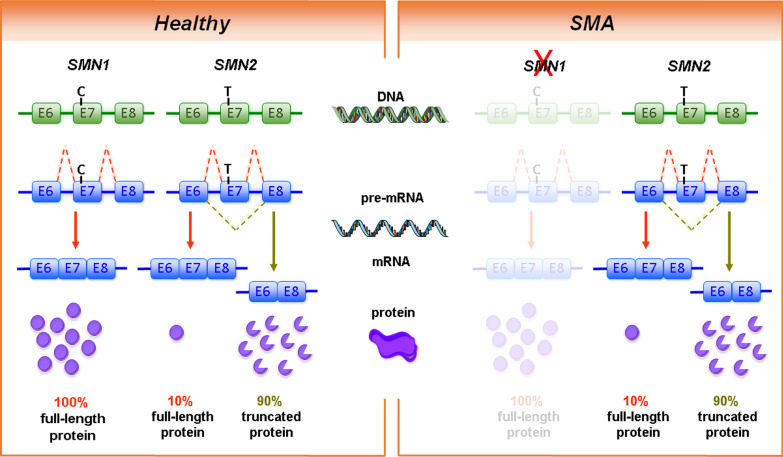
Genetic cause of spinal muscular atrophy (SMA). The human survival motor neuron genes (*SMN1 and SMN2*) are located in an inverse duplicated region on chromosome 5q13.2. On DNA level, the two genes only differ by one functionally relevant nucleotide exchange within exon 7. This transition from C to T results in the generation of an exonic splicing silencer (ESS) site leading to exon 7 skipping on mRNA level. While *SMN1* codes for the functional, full-length SMN protein, *SMN2* predominantly (~ 90%) produces a truncated, non-functional version of the protein. SMA is caused by homozygous deletions of *SMN1* resulting in highly reduced SMN protein levels. However, the number of *SMN2* copies that determines the amount of functional SMN protein can modify disease severity

Humans carry two *SMN* genes (*SMN1* and *SMN2*) within a duplicated region on chromosome 5q. Homozygous loss or mutations of *SMN1* cause SMA, whereas loss of *SMN2* is usually not associated with the disease. During evolution, the duplication of the *SMN* gene occurred at the stage of non-human primates [251]. In laboratory mice and other rodents, the *Smn* gene is not duplicated [263, 264]. *SMN1* and *SMN2* differ only in a few nucleotides. Of particular importance is the C to T transition in exon 7 of the centromeric *SMN2* which causes alternative splicing of exon 7. Most transcripts from the *SMN2* gene lack exon 7-encoded domains, resulting in only 5–10% full-length SMN protein in comparison to 100% full-length SMN protein from *SMN1* transcripts (Fig. 1)*.* Therefore, *SMN2* can only partially compensate for *SMN1* loss [180, 206, 207]. Most SMA patients carry 2–3 *SMN2* copies. This allows cellular production of approximately 10–30% full-length SMN protein in comparison to healthy controls with intact *SMN1* gene copies. Thus, the *SMN2* copy number is the most important genetic modifier of SMA disease severity [85, 319].

The majority of the severely affected SMA patients bear homozygous deletions of *SMN1* whereas most SMA type 2 and 3 patients show a homozygous absence of *SMN1* due to a gene conversion of *SMN1* into *SMN2 *[37, 318]. Gene conversion is a common cause for *SMN2* gene copy number variations, increasing the *SMN2* gene copy number from 2 to 3 or 4 [40]. Four copies of *SMN2* usually generate sufficient functional SMN protein for a milder disease phenotype [85, 185] in SMA type 3 patients.

In about 5% of SMA patients, point mutations are detected in the *SMN1* gene mostly in exon 6 and 7 [320]. Such cases are termed “compound heterozygotes”—with a deletion/conversion in one allele and a point mutation in the other.

Apart from 5q-SMA, other forms of spinal muscular atrophies exist which can be classified into the following categories on the basis of disease phenotype and genetic inheritance: autosomal recessive and autosomal dominant distal spinal muscular atrophies (DSMAs); autosomal dominant proximal spinal muscular atrophies; autosomal recessive non-5q spinal and bulbar muscular atrophies; X-linked recessive SMAs.

### Genetic modifiers in SMA

A transcriptome-wide differential expression analysis of total RNA from lymphoblastoid cells, derived from *SMN1*-deficient siblings with discordant disease phenotype, revealed a significant association between disease severity and Plastin 3 (PLS3) expression [224]. *PLS3* maps to Xq23 [282]. The gene is located on the X-chromosome and appears as a sex-specific modifier of SMA. Plastins are evolutionarily conserved and function as modulators of the actin cytoskeleton. Thus they play an important role in cell migration, adhesion and exo- and endocytosis [321]. Additional genetic modifiers in SMA include Neurocalcin delta (NCALD) and Calcineurin-like EF-hand Protein 1 (CHP1). Both proteins act as Ca^2+^-sensors and Ca^2+^-binding proteins [124, 143, 246]. All three SMA protective modifiers are not active in the assembly of spliceosomal snRNPs. Since they are involved in modulating various cellular processes including the rescue of impaired endocytosis in Smn-deficient cells and animal models [64, 124, 143, 246], these SMA modifiers turned into potential therapeutic targets.

### The cellular and molecular function of SMN

The SMN protein is ubiquitously expressed and not only found in the nervous system especially in motoneurons. SMN acts in a protein complex that mediates spliceosomal snRNP assembly [91, 176, 194, 233]. Classical *Smn* gene knockout in mice causes early embryonic lethality [264], which is consistent with a fundamental role of Smn in all cell types as an essential cellular protein for pre-mRNA processing. Mice with homozygous gene knockout of endogenous *Smn* with 2 additional transgenic copies of human *SMN2* develop severe SMA, thus mimicking SMA type 1 in humans [208]. However, mRNA levels in most organs of these mice including brain appear normal, and splicing of defined transcripts is unaffected [140]. This indicates that processing of pre-mRNAs including splicing in general is not affected in SMA. However, it cannot be excluded that few transcripts require high levels of SMN protein and SMN complex so that the levels of SMN that can be produced from up to four copies of *SMN2* are not sufficient in such cells. Studies in Drosophila have provided evidence that specific transcripts requiring the U11/12 minor splice complex appear more vulnerable to Smn depletion [132, 177, 181] than the majority of transcripts that are processed via the U1, 2, 4, 5, 6-dependent major spliceosome complex. However, these findings have been challenged by the observation that development of Smn-deficient flies in general is delayed and that U11/12-dependent pre-mRNA splicing during normal development occurs only at later larval stages [98]. Thus, the lower levels of U11/12 minor splice complex-dependent mRNA modifications could reflect a delay of larval development in Smn-deficient flies. Although U11/12 as well as U2-dependent intron retention have been observed in transcripts in Smn-deficient flies and mice, only few were reproducibly confirmed, such as TMEM41B/Stasimon and Mdm2/4 [66, 181, 275, 300]. Despite the observation that the restoration of Mdm2/4 expression improved motor functions to some degree, this restoration of Mdm2/4 did not beneficially affect survival of SMA mice [300]. Beside the components of the classical SMN complex, additional SMN interaction partners have been identified; among them hnRNP R [256], TDP-43 [296], FUS [323] and HuD [82], which are involved in many neuronal functions including transcription regulation, nuclear pre-mRNA processing, nuclear export and subcellular transport of many mRNAs [4, 18, 81, 82, 102, 104, 110, 218, 255, 325, 326]. In particular the axonal translocation of the β-actin mRNA is severely disturbed in Smn- [255], hnRNP R- [102] and TDP-43- [33] deficient neurons. HnRNP R as an interaction partner of SMN is found in the nucleus and the cytosol, including axons of motoneurons [68, 256]. It is involved in subcellular transport of mRNAs and other types of RNA in axons [32, 34, 261].

### Regulation of SMN expression during development

The developmental expression of SMN in mice and humans shows unique dynamic features. SMN protein levels are high during prenatal development and decline during early perinatal stages [20, 38, 97, 140, 144, 240, 241]. In blood, higher SMN expression levels are found in young children compared to adults [309, 330]. The median SMN protein level was 2.3-fold higher in prenatal healthy individuals in comparison to early postnatal children younger than 3 months. This difference increases during development. SMN protein levels are about 6.5-fold reduced in human autopsy tissue samples (lumbar or thoracic spinal cord) in individuals aged 3 months through 14 years [240, 241] when compared to samples from fetal stages. SMN levels are fourfold lower in human spinal cord samples from SMA patients at postnatal stages (up to 3 months of age) in comparison to healthy subjects. Downregulation of the high prenatal SMN protein levels at early postnatal stages was also observed in frontal cortex, diaphragm and skeletal muscles [240, 241].

SMN protein levels correlate only modestly with total *SMN1* and *SMN2* mRNA transcript levels in prenatal tissue samples. The decline in median *SMN1* full-length or *SMN2* mRNA levels at early postnatal stages in tissues from healthy controls is mild in comparison to the protein level [240, 241]. This indicates that SMN protein levels decline at early postnatal stages independent from *SMN* promoter activity [71, 206, 207, 257] via posttranscriptional mechanisms [53, 149]. In mice, Smn protein levels decline in spinal cord between embryonic day (E) 14 and 19. This period is followed by a further decline between postnatal day (P) 5 and 15 [140]. At the moment, the mechanisms which regulate SMN expression at posttranscriptional and posttranslational levels are not fully resolved. Both, translational control and the regulation of Smn protein degradation could play a role. The instability of SMNΔ7 protein is mediated by a degradation signal termed degron (SMNΔ7-DEG) which is created by the new C-terminus in the truncated protein from the *SMN2* gene [45]. Inactivation of SMNΔ7-DEG by a point mutation stabilizes SMNΔ7, which in turn is able to compensate for SMN loss in cell lines.

### SMA: motoneuron and neuromuscular pathology

As stated before, relative SMN protein levels are highest at prenatal stages in humans and in mice (1 week before birth) [140, 240, 241] implying a crucial role of SMN in cellular differentiation. In motoneurons, this period of high SMN protein levels coincides with the developmental stage when these neurons grow out axons and form synaptic contacts with skeletal/striated muscle fibers to establish neuromuscular endplates. These findings suggest that high amounts of SMN protein are necessary for proper development of the neuromuscular system [38, 140, 141]. During early prenatal development, about half of the postmitotic motoneurons that have been originally generated in the spinal cord undergo physiological cell death. Developmental cell death is controlled by neurotrophic factors [11, 116, 129, 234, 265, 266, 268]. In humans as well as in mice, SMN deficiency does not amplify motoneuron loss during this critical developmental period. This developmental period of physiological motoneuron death is followed by a ssynapses are eliminatedtage when supranumerary synapses are eliminated [173], so that one muscle fiber receives synaptic input only from one motoneuron. This time window of polysynaptic elimination coincides with deterioration of motor function and motoneuron degeneration at least in mouse models of SMA type 1 and 2 [103, 126, 166]. During this stage, about 17–29% motoneurons are lost in SMA type 1 mouse models in comparison to healthy littermates [208]. Motoneuron loss continues after these early postnatal stages. In heterozygous *Smn* mice in which only one allele of *Smn* is deleted, resulting in a reduction of 50% Smn protein levels, about 50% of motoneurons are lost at a stage of 12 months [140, 274]. Likewise in children with SMA type 1, severe motoneuron loss has been observed at disease endstage. At an age of 5–22 months, motoneuron loss in patients with type 1 SMA increases to more than 70% [272].

When motoneurons are isolated from embryonic *Smn*^*−/−*^*;SMN2* mice and cultured for periods up to 7 days, cell death is not enhanced but axon extension is markedly altered. This axonal defect [139, 156, 255] appears as a prominent feature and is also observed in other Smn-deficient animal models such as zebrafish [192, 317]. Defective axon growth correlates with reduced actin dynamics [211, 255] and altered excitability through voltage-gated Ca^2+^-channels [139]. Treatment of SMA and control mice with the calcium channel modulator R-Roscovitine results in an increased number of preserved and even regenerating neuromuscular junctions (NMJs) [291]. Thus, defective presynaptic activity and reduced transmitter release apparently contribute to pathology and degeneration of neuromuscular junctions and axons in SMA.

A common and characteristic pathological feature of SMA is that proximal muscle groups appear more vulnerable than distal muscles. For example, muscle groups for finger movements appear less affected than the trapezius, deltoid, quadriceps or gastrocnemic muscles [69]. This appears on a first view counterintuitive because motoneurons with long axons are generally considered to be more vulnerable than those with shorter axons. However, motoneurons that innervate muscle groups for position control usually generate large motor units with highly branched motor axons up to several thousand terminals. In contrast, motor units for fine movements of fingers or posture are usually small. For example, the motor unit in finger muscles such as the first lumbrical muscle is in a range of 100 [84, 115]. In contrast, the gastrocnemius muscle has an innervation ratio of 1000–2000 muscle fibers per motoneuron [84]. Thus, vulnerability of motoneurons in SMA seems to correlate with the size of motor units. Axons of motoneurons contain relatively high levels of mRNAs which are transported into these distal neuronal compartments, where they are locally translated [34, 220, 261]. Transcripts encoding actin, mitochondrial proteins or components of presynaptic active zones are highly enriched in motor axons. The transport of these transcripts seems to be highly disturbed in Smn-deficient motoneurons [34, 211, 261]. To some extent, defective translocation of transcripts for these proteins seems to be compensated in motoneurons with low numbers of axonal branches and corresponding low numbers of neuromuscular junctions that are served by these branches. However, in motoneurons of large motor units such compensatory processes might be limited, and then probably leading to degeneration of presynaptic compartments and retrograde degeneration of axons.

### Muscle pathology in SMA

Motoneuron and skeletal muscle maturation closely correspond and depend on cellular contact between each other. Although muscle atrophy in SMA is primarily caused by denervation, there is growing evidence that muscle-autonomous alterations also occur in SMA and could contribute directly to disease pathogenesis. The Smn complex is localized at sarcomeric z-discs in striated myofibrils [311]. Muscle-specific *Smn* knockout in mice without reducing Smn expression in motoneurons results in massive muscular dystrophy [47], implying a cellular function of the Smn protein also in the skeletal muscle. Similar observations were recently reported by Kim et al. [151], as muscle-specific Smn depletion in mice induces morphological alterations in myofibers and NMJs. This correlates with reduced ex vivo force and impaired motor function by 6–7 months of age and reduced lifespan [151]. Other studies on selective restoration of Smn protein expression by 50% in muscle satellite cells showed significant improvement of the SMA phenotype in mice [219]. The hypothesis of muscle-specific disease mechanisms has also been discussed on the basis of morphometric studies of human SMN-deficient skeletal muscle samples [22, 87, 187]. Initial studies, performed more than 20 years ago, already suggested that type 1 and 2 (but not type 3) SMA patient-derived myofibers degenerate after one to three weeks in co-culture with wild type fetal rat spinal cord explants [30]. This indicates that myoblasts and myofibers from type 1 and type 2 SMA patients have a higher vulnerability and propensity to undergo cell death. Abnormal expression of markers for skeletal muscle development such as slow and fast myosin [186] and defective myoblast fusion have also been reported [12, 31, 114, 190, 271]. Hence, these findings indicate that myogenesis is delayed in SMA.

Taken together, these data indicate that expression of SMN in skeletal muscles is important for proper development and maintenance, and that SMN levels should not fall below a threshold that so far is not well-defined. The data from Braun et al. [30] suggest that SMN protein levels might be sufficient in SMA type 3 but not type 1 SMA, although other studies on SMN in muscle reported contradictory effects [99, 137]. Muscle-specific overexpression of Smn in the severe *Smn*^*−/−*^*;SMN2* mouse model showed no beneficial phenotypic effect [99]. Second, a muscle-specific *Smn* knockdown—via Myf5-Cre and the Cre-loxP recombination system—on a *SMN2/SMNΔ7* background did not cause any SMA symptoms [137]. The extensor digitorum longus muscle was investigated for this study [137], a muscle which is usually not heavily denervated in the *SMNΔ7* mouse model [174]. However, these findings support the hypothesis that SMN thresholds could play a crucial role in different muscle types depending on the severity of the disease. It is still unclear how the SMN expression levels change in later life. It could be that the expression level in muscle also falls below a critical threshold when SMN expression decreases during later postnatal development.

This could lead to a situation that a myopathic disease mechanism could contribute to the disease phenotype or even pre-dominate neurogenic muscle atrophy in milder SMA types. SMN protein levels in muscle in general are much lower than in spinal cord or brain [50, 138], and these levels are also expected to decrease during life time. Relevant data from SMA patients to judge the extent of altered SMN protein expression during life are missing. This raises the point that ubiquitous SMN upregulation via a therapeutic approach with a small molecule such as Risdiplam might counteract the myopathic phenotype better at higher age than a therapeutic approach delivering the drug through intrathecal administration only to spinal cord and brain.

#### Muscle pathology in non-5q-SMA

Spinal muscular atrophy with respiratory distress type 1 (SMARD1) also referred to as DSMA1 (distal spinal muscular atrophy type 1), is a fatal motoneuron disorder which usually starts in infancy and early childhood [23, 109]. SMARD1 is characterized by dysfunction and progressive degeneration of motoneurons in the ventral horn of the spinal cord, resulting in a neurogenic atrophy of striatal and skeletal muscle fibers [107]. In addition, diaphragm and heart muscle are primarily affected in the mouse model. This results in a mixed phenotype comprising both primary and neurogenic muscle degeneration. Muscle weakness in SMARD1 patients predominantly affects distal muscle groups, usually starting in the lower limbs. The most prominent and defining symptom of SMARD1 is a life-threatening respiratory distress due to a severe paralysis of the diaphragm [107, 108, 259]. The neuromuscular degeneration (*Nmd*^*2J*^) mouse is a model system for the juvenile form of SMARD1 [51]. The pathological features of the *Nmd*^*2J*^ mouse are comparable to humans. However, muscle fiber degeneration in the diaphragm does not correlate with motor axon loss in the phrenic nerve [106, 158, 307]. That means there is a clear distinction between motoneuron degeneration and myopathy in this form of spinal muscular atrophy.

## Approved therapies for SMA

**Fig. 2:**
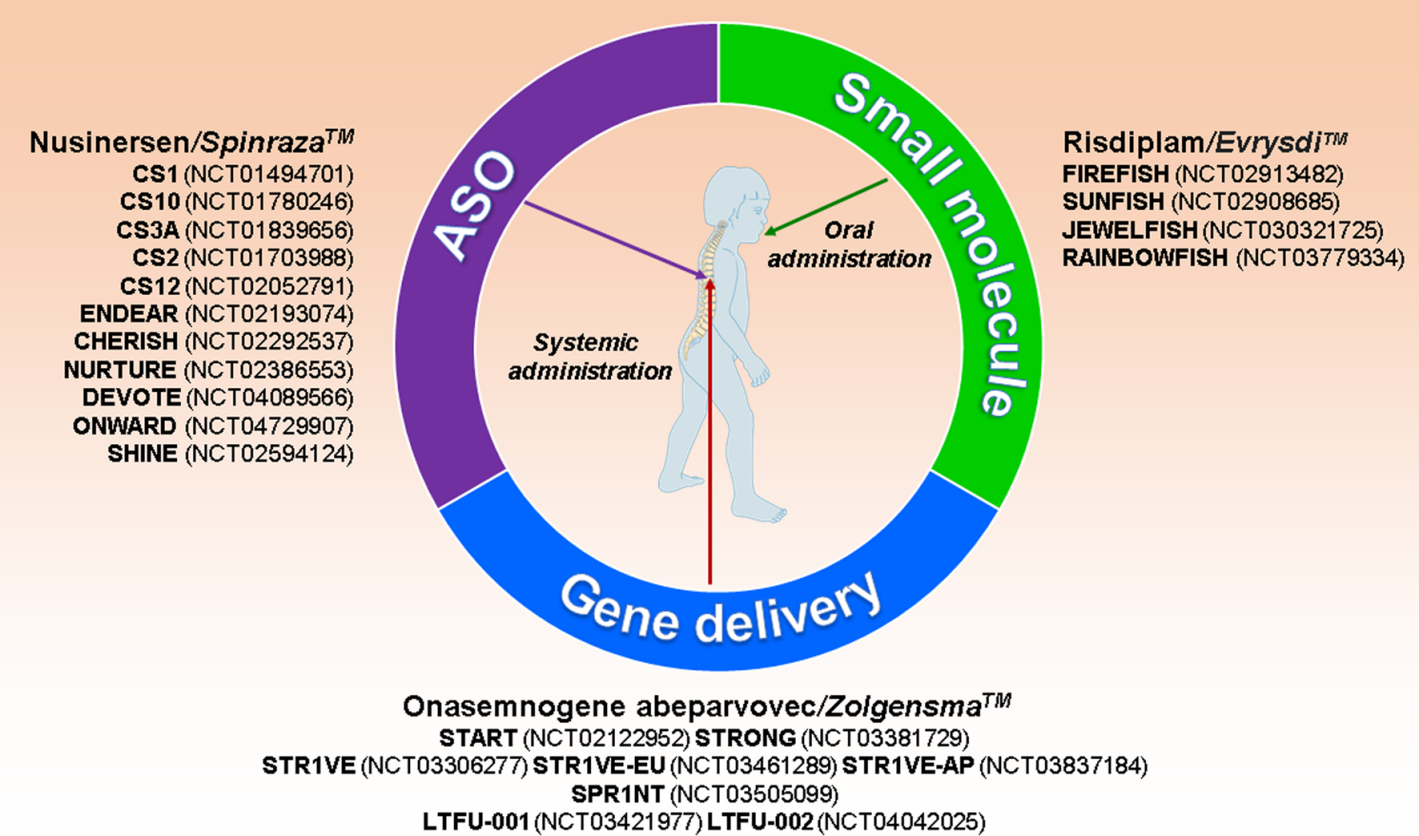
Gene therapies in SMA. Overview of the currently available therapies for spinal muscular atrophy (SMA): Antisense Oligonucleotide (ASO: Nusinersen), small molecule (Risdiplam), AAV9 gene delivery approach (Onasemnogene abeparvovec)—and corresponding clinical trials

Significant advances in basic research and clinical studies paved the way for FDA- (Food and Drug Administration, USA) as well as EMA- (European Medicines Agency, EU) approved SMA therapies. All of these approved therapies focus on strategies aiming at increased SMN protein expression, either by modulation of *SMN2* splicing to increase exon 7 inclusion via Antisense Oligonucleotides (ASOs: Nusinersen/Spinraza™) or small molecules (Risdiplam/Evrysdi™), or by viral gene transfer for introduction of an intact additional *SMN1* cDNA copy (Onasemnogene abeparvovec/Zolgensma™) (Fig. 2). Figure 2 summarizes all studies which are described and discussed in more detail hereinafter. More information can be obtained from https://clinicaltrials.gov. All therapies have been developed in mouse models for SMA, and their efficacies have been optimized with established cell culture models. They thus stand as a success story for translational research from basic cellular and molecular neuroscience models towards new therapies for treatment of a neurodegenerative disease.

At present, there are three different therapies for SMA approved by the FDA and the EMA that use different strategies to enhance functional, full-length SMN protein levels. Antisense Oligonucleotides (ASOs), known under the name Spinraza™, are intrathecally injected and are designed to inhibit exon 7 skipping in *SMN2* transcripts. Small molecules such as Evrysdi™ are applied orally and modulate exon 7 inclusion similarly to ASOs. The third treatment option is gene therapy via systemic intravenous application of a non-replicating self-complementary adeno-associated virus 9 (scAAV9) that introduces *SMN1* cDNA (Zolgensma™) into infected cells.

### Antisense oligonucleotide strategies

#### Nusinersen/Spinraza™: an ASO-approach

ASOs are short strands of synthetic nucleic acids which bind target-RNA by complementary base pairing to modulate RNA stability, structure and function [247]. Nusinersen is an 18-mer ASO modified by 2‘-*O*-2-methoxyethyl phosphorothioate to protect it from rapid degradation. It was designed to block the binding of hnRNP A1 to the intronic splicing silencer N1-(ISS-N1) motif in intron 7 of the *SMN2* gene. The block of hnRNP A1 binding to this domain in turn disrupts a splice inhibitor site and thus promotes exon 7 inclusion in the pre-mRNA that is derived from the *SMN2* gene [128]. Due to their size, ASOs cannot cross the blood–brain barrier (BBB) and have to be applied by intrathecal administration so that they can be taken up by motoneurons from the cerebrospinal fluid (CSF).

Nusinersen was the first drug that has been approved for the treatment of SMA by the FDA in December 2016 and by the EMA in June 2017. Currently 31 clinical trials have been reported by https://clinicaltrials.gov. Herein we report a selection of studies focusing on dose finding with already disclosed data. The first phase 1 clinical trial with Nusinersen (CS1, NCT01494701 and CS10, NCT01780246) was conducted with 28 patients (2 to 14 years of age) with SMA type 2 and type 3. This study provided evidence that intrathecal delivery of a single dose of Nusinersen (1 mg, 3 mg, 6 mg, or 9 mg) is safe and well tolerated. Nusinersen more than doubled SMN protein levels in the CSF in the 6 mg and 9 mg treatment groups. This was accompanied by a significant increase in motor function illustrated by the Hammersmith Functional Motor Scale Expanded (HFMSE) scores in the 9 mg group [44]. A subsequent phase 2, open-label study (CS3A; NCT01839656) with SMA type 1 patients with 2–3 *SMN2* copies (3 weeks to 6 months of age) was conducted with multiple doses of Nusinersen. Four patients received ascending doses of 6 to 12 mg and 16 patients a 12 mg intrathecal injection. The patients in the 12 mg group exhibited incremental achievements in developmental motor milestones on the Hammersmith Infant Neurological Examination-2 (HINE-2) score (from baseline to last visit p < 0.0001), improvement in Children’s Hospital of Philadelphia Infant Test of Neuromuscular Disorders (CHOP INTEND) motor function scores (p = 0.0013) and significantly increased CMAP for abductor digiti minimus and tibialis anterior via stimulation of the ulnar nerve or the peroneal nerve. Probability of permanent ventilation-free survival was also significantly increased. Examination of post mortem tissue revealed an even distribution of Nusinersen throughout spinal cord, including motoneurons and brain that coincided with an enhanced exon 7 inclusion in *SMN2* and an increase of the SMN protein [88]. Subsequent studies with Nusinersen treatment in 28 patients with SMA type 2 and type 3 (age 2—5 years) for approximately 3 years showed a long-term benefit (CS2; NCT01703988 and CS12; NCT02052791). The patients started with ascending doses (3, 6, 9, 12 mg over 253 days) followed by a period of treatment with 12 mg every 6 months for more than two years (CS12) [54]. This was followed by the SHINE study (NCT02594124) via continuing 12 mg Nusinersen applications to assess the long-term clinical effects of Nusinersen. The treatment over the first three years resulted in motor function improvements and disease activity stabilization that differed significantly from the natural disease history. Participants with later-onset SMA in CS2/CS12/SHINE displayed increases in walking distances that were not observed in natural history cohorts [210] with stabilization in fatigue and improvements of ambulatory function over the period of Nusinersen treatment (~ 5.5 years) [209]. The phase 1/2 results encouraged the design of two large, multicenter, randomized, sham-controlled, phase 3 studies with Nusinersen: ENDEAR (NCT02193074) in SMA type 1 patients and CHERISH (NCT02292537) in SMA type 2 patients. The ENDEAR study (NCT02193074) included 122 SMA type 1 patients at 7 months of age or younger. They were randomized to receive multiple intrathecal doses of Nusinersen or a sham procedure at a ratio of 2 to 1 [89]. In the CHERISH study (NCT02292537) 126 children were randomly assigned, in a 2:1 ratio, to receive multiple doses of 12 mg Nusinersen or a sham procedure. The median age at study onset was 4 years (2–9 years) in the Nusinersen group and 3 years (2–7 years) in the control group [196]. In both studies, treated children showed a significant improvement in motor function compared to control groups. In the ENDEAR study the overall survival was higher in the Nusinersen-treated group than in the control group. A very striking observation was made, as infants with shorter disease duration at the study onset were more likely to benefit from Nusinersen than those with longer disease duration. The crucial timing of initiation of Nusinersen treatment for maximal therapeutic benefit is currently under investigation in a phase 2 study of pre-symptomatic patients (NURTURE, NCT02386553). The 25 included patients are still alive and do not require permanent ventilation. All patients are able to sit without support and achieved walking with or without assistance and still without ventilation support [59]. In December 2016 Nusinersen/Spinraza™ became available at a recommended dose of 12 mg per treatment for all patients. Currently, safety and efficacy of higher doses are in focus of the DEVOTE study (NCT04089566). DEVOTE is subdivided into part A, B, C. Part A is an open-label study focusing on safety and tolerability of Nusinersen (3 × 28 mg loading doses and 2 × 28 mg maintenance doses). Part B should demonstrate that higher doses improve participants’ outcomes measured by CHOP INTEND and motor skill ability. This part is designed as a randomized, double-blind, active-controlled study with infants and later-onset SMA patients. Patients will receive 4 × 12 mg loading doses, followed by 2 × 12 mg maintenance doses or 2 × 50 mg loading doses and 2 × 28 mg maintenance doses. Participants receiving the FDA-approved 12 mg of Nusinersen will serve as controls. The open-label part C will evaluate the safety and tolerability of transitioning patients that have already been treated with Nusinersen for at least one year. They will receive a single initial 20 mg dose followed by two 28 mg maintenance doses at four and eight months after therapy onset. The DEVOTE trial will then be followed by the open-label extension study ONWARD (NCT04729907) as a long-term extension.

Expanded access programs (EAPs) for Nusinersen had been initiated in several countries to verify therapeutic benefit with motor function improvements [10, 83, 100, 199, 232]. Two studies on SMA patients with adult onset (mean age 16–65 and 18–72) have been recently reported by Hagenacker et al., and Maggi et al. [111, 184]. The primary outcome in both studies was an increase of the HFMSE score. Maggi et al. additionally reported that RULM (Revised Upper Limb Module) score improved significantly in sitters [184]. Both studies provide evidence of Nusinersen safety and efficacy in SMA type 2 and type 3 patients.

In some patients, hydrocephalus has been reported as a potential side effect [10, 83, 100, 199, 232, 287]. The most commonly occurring side effects include lower respiratory infection and constipation in SMA infants whereas headache, vomiting and back pain are also observed in SMA patients with later onset (summarized in https://www.drugs.com/sfx/nusinersen-side-effects.html).

In SMA patients with significant scoliosis or patients who had received surgical spinal fusion, the intrathecal application of Nusinersen is challenging. It usually requires the use of computer-tomography guidance, video fluorangiography, ultrasound, or alternative administration techniques such as subcutaneous intrathecal catheters [199, 283, 314]. However, such new devices for Nusinersen administration have not been approved so far by the relevant regulatory authorities.

For unknown reasons, some patients respond better to the ASO than others [54]. A problematic issue with Nusinersen/Spinraza™ is the lack of systemic availability and the potential lack of efficacy to counteract long-term adverse effects of low SMN levels in peripheral tissues. Nusinersen restores SMN expression only in the central nervous system. There are preclinical data indicating that restoration of SMN protein levels might also be important for peripheral tissues such as liver, kidney, muscle and heart [112, 127].

### The small molecule and splicing modifier Risdiplam/Evrysdi™

Another option to restore SMN protein levels through increasing exon 7 inclusion in *SMN2* transcripts is via small molecules. Such small molecules appear of advantage especially when they can cross the BBB. When administered systemically, they then could act on the processing of the *SMN2* gene transcript in peripheral organs. Such small molecules have also been shown to modulate *SMN2* splicing. They are bioavailable after oral administration and distributed systemically, thus targeting not only the central nervous system but also the peripheral nervous system and non-neuronal organs and tissues [217, 242, 243]. A potential disadvantage of the small splice modifiers in comparison to ASO-based drugs is the higher propensity for off-target effects [28]. To bypass such unspecific effects, a high throughput screening for *SMN2* splicing modifiers was performed to receive optimal candidates such as RO073406/RG7916 (Risdiplam) [217, 237, 242]. Risdiplam increases SMN protein levels not only in CNS but also in peripheral tissues in two mouse models of SMA [237]. This effect is achieved by stabilizing the U1:5’ss duplex at the 5’ss of *SMN2* exon 7 [39, 227, 276]. Nevertheless, Risdiplam still produces off-target effects on splicing of exons of several other transcripts such as those coding for STRN3, FOXM1, APLP2, MADD, SLC25A17 [242, 276]. Administration at 1 mg/kg of body weight produces a robust enhancement in SMN levels in brain and quadriceps muscle in a SMA mouse model. It counteracts NMJ pathology and reduces motoneuron loss [242, 243, 276]. Higher levels of Risdiplam (10 mg/kg body weight) improve life expectancy in SMA mouse models to the same level as for healthy littermates [217].

On the clinical level, the evaluation of safety, tolerability, and efficacy of this drug was tested in SMA patients in the FIREFISH trial (SMA type 1 patients, NCT02913482) and the SUNFISH trial (SMA type 2 and 3 patients, NCT02908685) [223]. The FIREFISH trial was designed for infantile-onset SMA as a two part non-randomized open-label study in which 41 patients (1–7 months) were enrolled and studied for one year. All patients had a homozygous deletion of *SMN1* gene and two copies of *SMN2*. 29% of patients were able to sit independently for at least 5 s after 12 months treatment, reaching relevant motor milestones, and 42% could live without permanent ventilation [55]. Treatment with Risdiplam caused an increase of SMN protein levels in blood [19]. SUNFISH is a two part trial with later-onset SMA patients (2 to 25 years), randomized and placebo-controlled. The first part with 51 participants is a dose-finding and safety tolerability study whereas part 2 with 180 SMA patients focusses on efficacy and safety [223]. The motor function skills of the Risdiplam-treated patients surpassed significantly those of the untreated patients after 24 months of treatment. No treatment-related adverse effects leading to withdrawal or treatment discontinuation during the 24 months trial period have been reported (Dr. Elizabeth Kichula CureSMA Meeting 2021; SUNFISH Part 2: Later-Onset SMA).

The JEWELFISH study (NCT030321725) was designed as a subsequent multicenter, open-label study primarily evaluating the safety and tolerability of once-daily oral administration of Risdiplam in SMA patients aged 6 months to 60 years who have previously enrolled in other studies including those with RG7800 (NCT02240355), Nusinersen, Olesoxime and Onasemnogene abeparvovoec [239]. The JEWELFISH population is heterogeneous with a broad spectrum of motor impairment at baseline. 174 SMA type 2 and 3 patients with 3 or 4 *SMN2* copies have been enrolled, including non-sitters but also walkers, some of them with scoliosis and hip subluxation or dislocation. No serious adverse events related to the drug was reported. No ophthalmological findings attributable to Risdiplam exposure were reported [270] as in preclinical studies with cynomolgus monkeys. Retinal toxicity was observed in these monkeys consisting of photoreceptor degeneration and microcystoid macular degeneration (MMD) in the central retina after 5–6 months of daily treatment [242]. On August 7, 2020 FDA approved Risdiplam (Evrysdi™) under the fast-track designation and rare pediatric disease priority review process [223, 276]. Data across SUNFISH, FIREFISH and JEWELFISH suggest that Risdiplam has a favorable safety profile. In accordance to this safety profile, the RAINBOWFISH trial (NCT03779334) focusing on pre-symptomatic SMA patients has been started but the enrollment of the patients is still ongoing. It is a multicenter open-label study to analyze efficacy, safety, and pharmacokinetics/dynamics of Risdiplam in infants aged from birth to 6 weeks. Risdiplam is orally administered once daily for 2 years followed by an open-label extension (OLE) phase of at least 3 years. A follow-up of at least 5 years for each participant enrolled will finalize the RAINBOWFISH study. In general, the most frequently observed adverse effects include fever, diarrhea, and rash. Especially in SMA infants, respiratory tract infections, pneumonia, bronchiolitis, hypotonia, constipation and vomiting have been observed (listed in https://www.drugs.com/sfx/risdiplam-side-effects.html).

Another small molecule termed Branaplam is still under investigation. Branaplam (previously known as LMI070) is a pyridazine derivative that interacts with *SMN2* pre-mRNA and enhances exon 7 inclusion to increase the level of functional SMN protein [227, 279]. Like Risdiplam, Branaplam can be orally administrated. Branaplam was originally expected to be tested in SMA type 1 infants in an open-label, two-part phase 1/2 study (NCT02268552). However, this study has not been initiated yet.

### *SMN1* gene-therapy: Onasemnogene abeparvovec/Zolgensma™

An alternative way to increase SMN protein levels in motoneurons and other cell types is the *SMN1* gene therapy. Since SMA is a monogenetic autosomal recessive disorder that is caused by loss of function of the SMN protein, it appears as an excellent target for gene therapies. The small *SMN1* cDNA can be easily packed into a non-replicating self-complementary (sc)AAV9 vector and delivered systemically. scAAV9 cannot only deliver the *SMN1* cDNA to muscle and other peripheral tissue, but also cross the BBB to reach the CNS and spinal motoneurons [92, 93, 195, 299]. Preclinical studies confirmed elevated SMN expression from AAV-mediated gene transfer in motoneurons and peripheral tissues in a mouse model of SMA [17, 93]. SMA mice that were treated with scAAV9-mediated *SMN1* gene therapy exhibited a significant extension of life span to over 250 days [93]. This successful viral gene delivery system for *SMN1* was termed as Onasemnogene abeparvovec. Onasemnogene abeparvovec commonly known as AVXS-101 is commercialized under the name Zolgensma™. Onasemnogene abeparvovec became the first gene therapy to be approved in the U.S. for the treatment of pediatric SMA patients (up to two years of age), and by the EMA. The recommended dose of 1.1 × 10^14^ vector genomes (vg) per kilogram (kg) body weight is delivered via a single intravenous injection. It appeared to be well tolerated in patients with SMA type 1 or 2 and pre-symptomatic SMA infants [125].

**Fig. 3:**
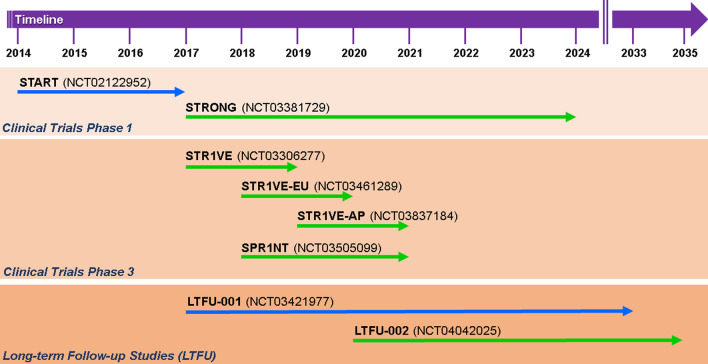
Timeline of the current Onasemnogene abeparvovec/Zolgensma™ trials. Illustration of the different clinical trials (clinical trials 1 and 3, LFTU) with Onasemnogene abeparvovec/Zolgensma™ according to their scheduled duration

Two clinical trials AVXS-101-CL-101 (START, NCT02122952) and CL-303 (STR1VE-US, NCT03306277; STR1VE-EU, NCT03461289; STR1VE AP, NCT03837184) were performed with symptomatic SMA type 1 patients carrying a two-allelic *SMN1* mutation and two *SMN2* copies [57, 58, 195, 198]. START was an open-label study with 15 SMA type 1 infants enrolled in two cohorts. Three infants were given a low dose of Onasemnogene abeparvovec (6.7 × 10^13^ vg/kg). All patients in the cohort (n = 15) were still alive without permanent ventilation at 20 months of age. In the high-dose cohort (12 patients, 1.1 × 10^14^ vg/kg), an increase from baseline of 9.8 points at 1 month and 15.4 points at 3 months in the CHOP INTEND score became detectable. In addition, 11 of the high-dose patients sat unassisted, 9 rolled over, 11 fed orally and could speak, and 2 walked independently. Elevated serum aminotransferase levels occurred in 4 patients and were treated by prednisolone [195]. The STR1VE-US study was an open-label, single-dose phase 3 trial. SMA patients with biallelic *SMN1* mutations (deletion or point mutations) and one to two *SMN2* copies were younger than 6 months and symptomatic. They received a single intravenous Onasemnogene abeparvovec dose of 1.1 × 10^14^ vg/kg body weight for 30–60 min. The monitoring of the 22 SMA patients was initially scheduled once per week and after 4 weeks once per month. All patients were able to sit independently for 30 s. 20 patients were free from permanent ventilation. All but one of the 12 patients in the high-dose cohort had gained significant motor milestones such as unassisted sitting, and serial incremental increases on the CHOP INTEND score of 50–60 points [57, 58]. Based on the data that had been collected in these trials, a long-lasting beneficial effect of Onasemnogene abeparvovec on motor function is expected. Adverse effects were bronchiolitis, pneumonia and respiratory distress. Only two patients showed elevated aminotransferases. The mechanisms of the immune and especially the hepatic response observed in these clinical studies are still not fully understood. One patient developed signs of hydrocephalus for which the mechanism is unclear. The CHOP INTEND results depicted early and fast benefits. The patients were able to thrive and swallow effectively without any cardiac pathology at the end of the study [57, 58]. Another clinical trial with Onasemnogene abeparvovec is ongoing as an open-label phase 1–2 trial with intrathecal administration in SMA type 2 patients (6 months to 60 months) with three *SMN2* copies (STRONG, NCT03381729). The STRONG study showed a sustained gain of motor milestones and treatment safety [90]. The intrathecal administration could circumvent potential immunological reactions in patients that had been pre-exposed to the virus before therapy. When an individual is exposed to endogenous AAV infections, an immune response can be initiated. [27, 312]. Thus a significant number of individuals produce neutralizing antibodies and block the gene transfer to cellular targets [3, 312]. The two studies by Day et al. [57, 58] revealed that 7.7% of the SMA patients and 14.8% of their biological mothers were positive for AAV9 reactive antibodies with exclusionary antibody titers > 1:50 on their initial screening tests. 5.6% showed elevated titers on their final screening and were excluded from receiving Onasemnogene abeparvovec in clinical trials. Therefore, the majority of SMA infants should benefit from Onasemnogene abeparvovec administration when given intrathecally [57, 58]. Another phase 3 study with intravenous administration in pre-symptomatic SMA type 1 or 2 patients with two or three copies of *SMN2* (SPR1NT, NCT03505099) has been initiated. Preliminary data suggest that Onasemnogene abeparvovec is well tolerated when applied at high concentrations (6.0 × 10^13^ vg/kg). Two long-term follow up studies (LTFU) are currently monitored until December 2033 and 2035. LT-001/NCT03421977 is the follow up safety study of 13 SMA patients type 1 in the AVXS-101-CL-101 trial (2017–2033), whereas LT-002/NCT04042025 (2020–2035) still enrolls patients from STRONG, STR1VE and SPR1NT. All clinical trials of Onasemnogene abeparvovec/Zolgensma™ along their timelines are summarized in Fig. 3.

### Temporal considerations for SMA therapies

Increasing the amount of SMN protein in neurons and other cell types does not only have advantages but also bears risks. The expression levels of SMN are highly controlled during normal development and cannot be fine-tuned by the gene therapy approach. Observations in mouse models argue that the viral overexpression could have long-term adverse side effects by interference with complex RNA processing mechanisms. In a recent study, it has been reported that long-term overexpression of AAV9-*SMN1* induces dose-dependent loss of proprioceptive synapses and neurodegeneration in SMA mouse models associated with loss of already achieved motor mile stones [301]. AAV9-*SMN1* leads to cytoplasmic SMN aggregation in neurons that corresponds to impaired snRNP biogenesis and widespread transcription abnormalities in DRG neurons [301]. These observations indicate that additional therapeutic targets other than *SMN1* and *SMN2* genes should be evaluated and considered (see below non-SMN treatments). For successful long-term treatment of SMA, it appears important to understand the time frames in which the SMN protein is needed to prevent disease development, and which cell types and organs need SMN at which time periods for proper homeostasis. SMN deficiency is embryonic lethal and SMA develops in early childhood or even prenatally within the critical time window when neuromuscular synapses are strengthened and become mature. The reconstitution of the SMN2 protein via an ASO approach during the early postnatal period in a severe SMA mouse model (P 1–4) was effective at preventing the onset of the disease. The survival rate of the treated SMA mice was prolonged up to 250 days of age [127] that corresponds to a 16-fold increase. Starting SMN elevation at 10 days after birth failed to deliver any benefit. Strikingly, the disease phenotype is reversible in milder SMA forms. In an intermediate SMA mouse model, post-symptomatic non-SMN treatment had beneficial effects [86]. However, delaying treatment for only one or a few days in severe SMA type 1 mice after symptom onset markedly lowered the benefit [93, 249] on motoneuron loss, and morphological alterations of neuromuscular endplates remained [183]. SMN protein deficits first impact the distal end of the motor unit [156, 208, 255]. Therefore, the optimal time window for reinstatement of SMN protein in severe cases depends on the maturation level of the neuromuscular endplates [147]. Studies with mouse models of SMA indicate that the optimal clinical effect in severe cases depends on early postnatal applications. This was also shown for SMN-independent therapeutic approaches in mouse models [291]. In two clinical trials with patients, similar observations were made. Starting the treatment pre-symptomatically in babies with two or three *SMN2* copies had significantly stronger effects than starting the therapy when first symptoms already occurred. When treatment with Nusinersen/Spinraza™ is initiated at less than 2 months of life, and after a median of 2.9 years of treatment, 100% of children could reach the milestone of sitting independently. Moreover, 88% patients could also walk independently [59]. In contrast, effects of therapy appeared markedly reduced in the phase 3 trial with Nusinersen/Spinraza™ where therapy was initiated after symptom onset in children with 2 copies of *SMN2*. Post-symptomatic treatment resulted in reduced mortality, but only 51% patients showed improved motor function and only 8% were able to sit independently at 13 months after therapy onset [89]. In conclusion, a delay of SMN elevation by several weeks up to 5 months can substantially reduce achievement of motor milestones. Therefore, it appears important that therapy in children with SMA starts immediately after diagnosis. These observations also provide a strong argument for systematic newborn screening, in order to detect pre-symptomatic and early symptomatic cases. Before symptoms appear and irreversible motoneuron degeneration starts, babies identified via newborn screening should be referred immediately to therapy. When SMN levels are very low, motoneurons lose their function within a short time frame and progress towards a stage when restoration might be limited, and the time window for successful restoration of motoneuron function and/or cellular regeneration could be missed.

The expenses for a Spinraza™ application in the first year amount to approximately $400,000–500,000 (or €400,000–500,000). In total $250,000–300,000 (or €250,000–300,000) per year are calculated for a patient’s lifetime. One single application of Zolgensma™ costs $2 million. In the near future an increasing number of gene-targeted therapeutic strategies are expected to be approved by the FDA and EMA. The costs for such therapies could then become problematic for health care providers.

### Non-SMN approaches

Enhanced synthesis of functional SMN protein via ASOs, small molecules or AAV9 vectors are current treatment options for SMA patients. However, with respect to the temporal requirements for SMN-based therapies, many SMA patients who cannot receive these therapies within an ideal time window will not fully recover and symptoms will remain or progress despite therapy, especially when the treatment starts at a later symptomatic stage. However, even when therapy starts at pre-symptomatic stages, not all SMA patients respond equally well and symptoms progress. In such cases, non-SMN approaches might support the SMN-based treatment strategies. Many of these non-SMN approaches target muscles, axons and presynaptic terminals at neuromuscular endplates.

#### Neuromuscular endplate

Neuromuscular endplates are severely affected in SMA. Not only reduced acetylcholine release [157, 260, 292], but also altered signaling mechanisms between motoneuron and muscle contribute to a degenerative process that ultimately results in muscle atrophy. Counteracting such signaling defects depends on (1) proper initial development of the neuromuscular endplates, including the presynaptic structures for controlled vesicle release, and (2) homeostatic mechanisms that maintain axons and presynaptic terminals in motoneurons. Primary cultured motoneurons from Smn-deficient mouse models have been used for characterization of the defective presynaptic compartment. Besides axon elongation alterations, defective F-actin assembly and reduced cluster formations of Ca_v_2.2, a voltage-gated calcium channel predominantly expressed in embryonic motoneurons, became apparent, leading to decreased spontaneous calcium transients [139, 211]. Dysregulated calcium influx due to disturbed cluster formations of Ca_v_2.1 which predominates in the neuromuscular endplate, have also been observed in vivo in mouse models of SMA [291, 292]. To sustain the required levels of presynaptic calcium for cellular differentiation and/or neurotransmission in Smn-deficient motoneurons, molecules that prolong the closing kinetics of Ca_v_2.2 and Ca_v_2.1 could be used as candidates. R-Roscovitine, a Cdk5 inhibitor with calcium channel modulating properties appears as a good candidate for this purpose [193, 324]. Acute R-Roscovitine application induces spontaneous Ca^2+^-transients in vitro and increases quantal content ex vivo. It improves motoneuron survival and expands life span of a severely affected mouse model after systemic treatment [291]. Histopathological analysis of R-Roscovitine-treated SMA mice revealed that application with this substance supports synapses in the spinal cord and counteracts degeneration of neuromuscular endplates [291]. In cultured Smn-deficient motoneurons, application of R-Roscovitine also rescued altered axon elongation. This effect was also observed by application of GV-58, another calcium channel opener [291]. GV-58 is more potent on Ca_v_2.1/2 and has less inhibitory activity for Cdk5 at physiological ATP levels [289]. It has already been tested in SMA mouse models where it showed significant benefits in terms of neuromuscular transmission and muscle strength [222]. However, both drugs also need to be administered during a critical period of NMJ development [308]. Thus, restoring intracellular Ca^2+^-homeostasis by external stimuli might be a therapeutic option for SMA, together with current ASO therapies, small molecules or adenoviral *SMN1*-gene transfer. Such kind of therapy needs also to be applied during a critical and early time window. Major recovery effects will not be obtained when treatment starts with delay and after symptom onset.

Substances with blocking properties of voltage-gated potassium channels such as 3,4-diaminopyridine (3,4-DAP) and 4-aminopyridine (4-AP) have also been discussed as candidates for increasing presynaptic calcium influx. On anatomical level, they increase the number of proprioceptive synapses projecting to motoneuron cell bodies in the spinal cord, as well as the number of NMJs in Smn-deficient mouse models. However, 4-AP treatment has no effect on motoneuron survival [273]. Clinical trials for both substances are ongoing (3,4-DAP, NCT03781479, NCT03819660 and 4-AP, NCT01645787).

In addition to these two molecules that modulate the kinetics of presynaptic voltage-gated Ca^2+^-channels, the effects of Pyridostigmine, an acetylcholinesterase inhibitor have been tested in SMA. This drug has been reported to increase fitness/perseverance in 2 of 4 SMA type 2 and 3 patients [310] (Clinicaltrials.gov: NCT02941328). Results from the last three clinical trials are still pending.

The genetic modifiers Plastin 3 and NCALD also act in a Ca^2+^-dependent manner in motoneurons. NCALD is a neuronal calcium sensor and functions as a negative regulator of endocytosis. NCALD knockdown improves endocytosis in SMA patients ‘ fibroblasts as well as axon elongation and neuromuscular morphology and function in SMA mice [246, 293]. However, the therapeutic potential of a NCALD knowdown needs to be further investigated, in particular with respect to toxicity and side effects. This appears important since MAP3K10 interacts with NCALD as an activator of c-Jun N-terminal kinases (JNKs). The activity of JNK is markedly upregulated in *NCALD*^*−/−*^ mice, probably affecting cellular differentiation, since morphology of NCALD-deficient hippocampal neurons is significantly altered [298]. Plastin 3 (PLS3), [224, 282] and other members of the Plastin family are evolutionarily conserved and act as modulators of the actin cytoskeleton. They play an important role in cell migration, adhesion and exo- and endocytosis [321]. In an Smn-deficient zebrafish model *Pls3* protein levels are reduced. However, *Pls3* mRNA-splicing is unaffected. A partial restoration of *Pls3* in these animals compensates presynaptic defects, independent of SMN expression [113]. *Pls3* orthologs are thus also considered as Smn modifier genes in Caenorhabditis elegans, Drosophila and mouse models [5, 65]. PLS3 appears as an interesting candidate for further therapeutic development, because of its regulation by Ca^2+^-ions in the presynaptic terminals of motoneurons, and its effect on actin bundling. These data and the observation of Ca^2+^-dysregulations in growth cones of Smn-deficient motoneurons in cell culture [139] and in vivo in neuromuscular endplates [260, 291, 292] support the hypothesis that Ca^2+^-dependent F-actin bundling could be a specific target for therapy development in SMA, in particular during early/prenatal development stages of the neuromuscular endplate.

#### Neuroprotection

Cellular differentiation of motoneurons depends on the presence and proper responsiveness to neurotrophic factors. It has been widely recognized that neurotrophic factor signalling contributes to motoneuron survival [11, 116, 129, 234, 235, 265, 268]. The application of brain-derived neurotrophic factor (BDNF), ciliary neurotrophic factor (CNTF) and/or glial derived neurotrophic factor (GDNF) to isolated primary motoneurons from chick, human, rat and Xenopus promotes their survival [116, 172, 266, 267], upregulates cholinergic differentiation and transmitter production [148, 322], and leads to increased acetylcholine release in quantal packets [175]. BDNF, as a member of the neurotrophin family, acts through the tropomyosin-related kinase (TrkB) family of receptor tyrosine kinases [152–154, 200, 281]. BDNF/TrkB signaling drives local calcium transients in mouse motoneurons cultured on synapse-specific laminin-221 [67]. The activation of TrkB downstream signaling cascades in turn promotes the stabilization of β-actin via the LIM kinase pathway and phosphorylation of Profilin at Tyr129 [67]. The specific effect of BDNF/TrkB signaling on on the cluster formation of presynaptic Ca^2+^-channels indicates that molecules which positively modify this signaling could beneficially act on this pathological aspect and improve neurotransmission in SMA. Neurotrophic factors and small molecules that activate specific pathways could also perform on other aspects of motoneuron pathology in SMA. Smn-deficiency leads to a downregulation of the Akt signaling pathway [295]. Loganin, a neuroprotective iridoid glycoside, has been described to upregulate BDNF and Akt signaling, resulting in improved motor function and mildly improved lifespan in SMNΔ7 mice [295]. IGF-1, a trophic factor that acts both on muscle, motoneuron development and survival, is reduced in severe SMA mouse models [215]. Systemic administration of IPLEX, a recombinant hIGF-1 complex with rhIGFBP-3, counteracts motoneuron degeneration and loss of motor function in SMNΔ7 mice, with minor effects on survival [215]. The overexpression of IGF-1 via systemic AAV1-mediated overexpression causes a slight upregulation of lifespan and improved motor coordination of SMNΔ7 mice [294]. Muscle-specific overexpression of IGF-1 through the myosin light chain promoter in SMNΔ7 mice had a positive effect on myofiber size. It also increases animal survival, but revealed no significant beneficial effect on motor function [26]. Nevertheless IGF-1 is still considered as an SMN-independent supplementary therapeutic approach. Likewise, Olesoxime (OLEOS, NCT02628743), a small orally active cholesterol-like molecule that targets components of the mitochondrial permeability complex, thereby preventing the apoptotic death pathways, has been tested. However, this drug candidate did not show convincing effects in clinical trials. At the preclinical level, Olesoxime preserves mitochondrial homeostasis and thus motoneuron integrity and reduces muscle denervation, astrogliosis, and microglial activation [25, 285]. However, the OLEOS clinical trials with type 2–3 SMA patients did not demonstrate any significant beneficial outcome.

#### Muscle-directed strategies

Muscle-autonomous disease mechanisms in SMA could contribute to the course of disease, in particular at later stages in SMA type 2 and 3 patients [50]. SMN levels in muscle and other tissues are very low in adult SMA mice and patients [50, 138]. In order to prevent loss of muscle mass, myostatin inhibition has been proposed as an option since this secreted growth/differentiation factor acts as a negative regulator of skeletal muscle fiber growth and size [169, 191, 332]. Myostatin activity is normally inhibited by follistatin and myostatin propeptide [118]. First studies with myostatin inhibition in models of severe SMA did not show significant effects [248, 284]. Interestingly, the effects of myostatin inhibition were stronger in mouse models of milder forms of SMA. In particular at later stages of disease, myostatin inhibition seems to have a positive effect on motor function and survival as well as muscle and bone atrophy [86, 179, 331]. SRK-015/Apitegromap is a selective monoclonal antibody that blocks myostatin [86, 236]. It is currently under investigation in the phase 2 TOPAZ study for 57 type 2 and type 3 SMA patients which already received Nusinersen (ambulatory cohort; 2–20 mg/kg). In the non-ambulatory cohort (20 mg/kg), it is tested both as a monotherapy and in combination with Nusinersen.

Along the same line, the troponin activator Reldesemtiv (CK-2127107) is considered as another modulator of muscle atrophy and loss of muscle strength. This molecule acts by slowing down calcium release from the troponin complex and thus sensitizes the sarcomere response to calcium [130], resulting in enhanced contractility. Most importantly, it amplifies the skeletal muscle force–frequency response upon nerve stimulation [9]. Reldesemtiv has been studied in a double blind, randomized, placebo-controlled, phase 2 study (NCT02644668) in two cohorts. Oral application of a single dose of Reldesemtiv has been well tolerated. Whether this treatment also improves motor function is currently investigated with larger cohorts of SMA patients [258].

Human Insulin-like growth factor 1 (IGF1), which has already shown neuroprotective potential in mouse models for SMA, was also tested in SMARD1/DSMA1 mouse models (*Nmd*^*2J*^ mouse). *Nmd*^*2J*^ mice are IGF-1-deficient and show an upregulation of IGF-1 receptor in gastrocnemius muscle and diaphragm, that is not observed in spinal cord [159]. The IGF-1 deficiency can be compensated by pharmacological application of human pegylated-IGF-1. This external application normalizes muscle fiber differentiation in the diaphragm and leads to a partial rescue in the gastrocnemius muscle [159]. Unfortunately, the compensatory effect of PEG-IGF-1 could not counteract atrophy. Cell body and axon loss of motoneurons is not diminished by IGF-1 treatment in *Nmd*^*2J*^ mice [159].

## Oligonucleotide and gene therapies beyond SMA for other neurodegenerative and muscular disorders

Most oligonucleotide therapies focus on gene silencing, transcriptional and splice modulation. Since oligonucleotides usually interact with their target molecules via complementary base pairing, gene-specific lead compounds can be derived from the primary sequence of the target gene. Also modifications for increasing bioavailability, such as for properly passing the plasma membrane and increased resistance to nucleases are feasible and have been successfully introduced [182]. In addition, bioinformatics tools allow avoiding predictable off-target effects. In terms of the ASO applications for individualized therapies, it is also possible to target patient-specific sequences in specific alleles such as single nucleotide polymorphisms (SNPs) or expanded repeat-containing mutant transcripts that are causative for rare diseases. This appears as an advantage over conventional screening for small molecules on the basis of effects on defined cellular target mechanisms. Although small molecules also bear the advantage of systemic application, they usually need extensive toxicological analyses and chemical optimization in order to lower off-target effects.

ASOs are classified into RNase H-competent ASOs and steric block ASOs without RNase H activity. Steric block oligonucleotides can interfere with transcript RNA–RNA and/or RNA–protein interactions and mask specific sequences within a target transcript [250]. They are mostly used for modulation of alternative splicing to exclude (exon skipping) or retain specific exon(s) (exon inclusion). In these cases, the oligonucleotide ‘masks’ a splicing signal converting it invisible to the spliceosome. This ultimately leads to alterations in splicing events e.g. for *SMN2* exon 7 retention [277]. In the following, we will give an overview of current ASO applications in neurodegenerative and muscular disorders beyond SMA.

### ASO therapies in amyotrophic lateral sclerosis (ALS)

Amyotrophic lateral sclerosis is a fatal motoneuron disorder, predominantly with adult onset. 10% of ALS cases are familial (fALS), whereas the remaining cases are considered ‘‘sporadic’’ (sALS) without a clear familial history [150]. Extended genetic analyses discovered all major genes for monogenetic forms of ALS [42]. However, the question concerning of how the relevant mutations affect the function of the corresponding gene products is in almost all cases not appropriately answered. This has consequences on the development of genetically based therapeutic strategies. In case such mutations cause loss of function (LOF), relevant approaches need to be designed for re-establishing this function. Gain of function (GOF) mutations would require the blockade/inhibition of the mutated gene and its product. However, downregulation of the expression of the mutated gene usually also affects expression of the unaffected allele. Most of the mutations described for the familial forms of ALS show dominant inheritance pattern and imply GOF mechanisms such as protein aggregates or altered protein properties affecting essential cellular processes [290]. However, there is evidence that GOF and LOF come together in some ALS-causing gene mutations such as the intronic expansion of *C9ORF72* [278, 290]. This situation then requires strategies that only affect the mutant gene and transcript. Herein we will exemplify ASO application in cases of *SOD1*, *C9ORF72,* and *FUS* and genetic ALS modifiers.

#### SOD1

The gene encoding Cu/Zn superoxide dismutase (*SOD1*) was the first identified mutation that causes fALS [62, 254]. The majority of the *SOD1* mutations (18.9%) correspond to fALS, while 1.2% coincide with sALS cases [334], both exhibiting a dominant inheritance pattern [6–8]. Most of the ALS-causing *SOD1* mutations do not reveal any correlation between SOD1 enzymatic activity and ALS disease severity [48, 221, 269]. Loss of function for Sod1 in *Sod1* gene knockout mice does not cause per se defects in motor axon elongation [142] or motoneuron degeneration [244]. In addition, the presence or absence of endogenous mouse Sod1 does not affect survival of mice expressing the human *SOD1G85R* transgene [36]. This argues for a toxic GOF although SOD1-LOF might not be completely excluded. In a recently completed placebo-controlled phase 1/2/3 clinical study, SOD1 was targeted by the ASO Tofersen/BIIB067 (NCT02623699 December 8th 2015 until March 24th 2021) for silencing the mutant as well as the wild type allele [201, 202]. This was a 3-part (A, B, C) study to examine efficacy, safety and tolerability of BIIB067. BIIB067 administration resulted in an approximately 36% suppression of SOD1 in the CSF which appeared safe for SOD1-ALS patients [201]. It remained open, whether this is sufficient for long-term suppression of toxic effects of mutant SOD1, although administration appeared safe. Only some lumbar puncture-related side effects have been reported [201]. The first two parts (phase 1/2) were primarily not designed for assessment of motor function. However, they revealed that this treatment possibly slows disease progression and leads to better performances in vital capacity and hand-held dynamometry tests [201]. These effects have been pursued in phase 3 started in May 2019 to evaluate clinical efficacy. All types of *SOD1* mutations and severity levels of symptomatic ALS patients were included. In order to explore therapeutic effects of BIIB067 in pre-symptomatic ALS patients, again a placebo-controlled phase 3 study (which is currently in the recruitment status) has been started on May 17th 2021 (NCT04856982). The primary objective is to evaluate efficacy of BII067 in pre-symptomatic ALS-SOD1 patients with elevated neurofilament levels. The secondary objectives include evaluation of safety, pharmacodynamics, and treatment-response biomarkers. The estimated study completion date is the second half of 2027.

#### C9ORF72

At least in Europe and the US, the most common genetic cause of both ALS and Frontotemporal Dementia (FTD) is a GGGGCC (G4C2) hexanucleotide repeat expansion in the open reading frame 72 (*C9ORF72*) gene localized on chromosome 9. The mutation constitutes approximately 34% of fALS and nearly 25% of familial FTD cases (C9ALS/FTD) in European populations [302, 334]. Up to 25 G4C2 repeats are found in healthy individuals, while C9ALS/FTD patients harbor hundreds to thousands of repeats [60, 245, 253]. The expansion is located in intron 1 which also contains the promoter region of the second transcript for *C9ORF72* [161, 204, 205]. Thus, reduced expression levels of the corresponding second transcript led to decreased C9ORF72 protein levels in such patients. However, this intronic expansion region is also translated via a non-canonical form of protein biosynthesis (repeat-associated non-AUG [RAN] translation) [15, 212, 335]. Based on the observations of RNA-foci or aberrant RNAs as well as the production of toxic homopolymeric dipeptide repeat proteins (DPRs) through RAN translation [94, 163, 205, 212, 286, 290, 315], a GOF mechanism appears to take place. Antisense RNA-foci are known to sequester RNA-binding proteins (RBPs), leading to LOF of RBPs in corresponding neurons [163, 168, 189]. Depletion of C9orf72 in isolated mouse motoneurons leads to alterations in axon growth and presynaptic differentiation [278]. This phenotype is also observed in C9ORF72 ALS patients' inducible pluripotent stem cell (iPSC)-derived motoneurons and resembles some of the alterations that are observed in cell culture models of SMA. Based on these findings, a combined therapeutic approach with silencing the G4C2-repeat-containing RNAs and simultaneous increase of C9ORF72 expression by gene therapy has been proposed for C9ORF72 patients [101]. The ASO BIIB078 that targets the sense-strand of *C9ORF72* transcripts containing the hexanucleotide G4C2 repeat has been tested for safety and tolerability in a phase 1 clinical trial with adult C9ORF72 ALS patients (NCT03626012). The study is still active with an estimated completion date end of 2021. The trial already starts to be followed by a phase 1 extension study (NCT04288856) to assess long-term safety, tolerability, pharmacokinetics and effects on disease progression of BIIB078 application to previously treated C9ORF72 patients. The study is still enrolling with an estimated completion in the middle of 2023.

#### FUS

**Fig. 4:**
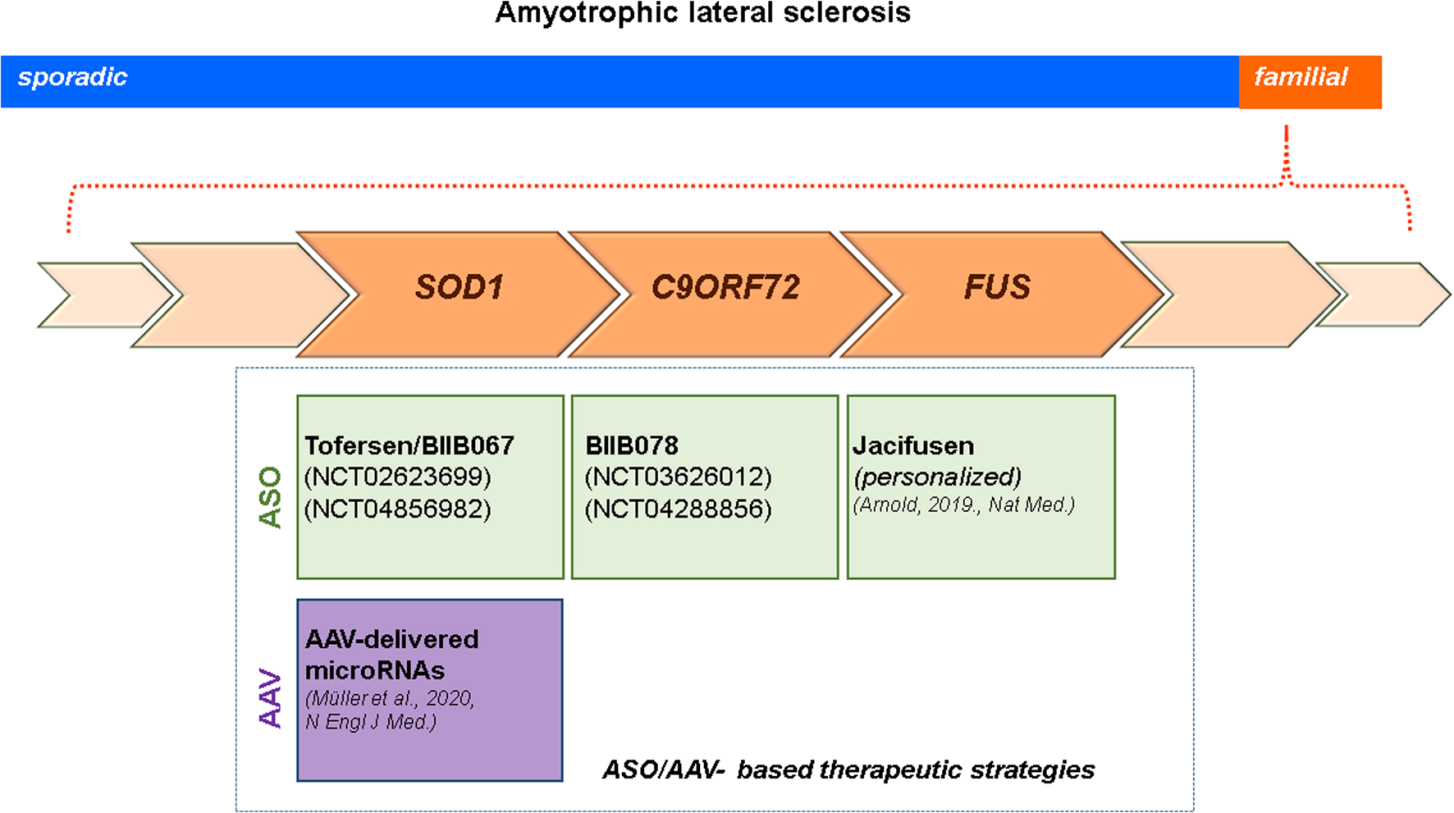
Different therapeutic strategies in familial forms of amyotrophic lateral sclerosis. Illustration of the different ASO and AAV approaches for the SOD1-, C9ORF72-, and FUS-ALS forms

Mutations in FUS/TLS (Fused in Sarcoma/Translocated in Liposarcoma) are a genetic cause for rare forms of fALS and FTD [35, 162, 303, 304]. *FUS* mutations are present in 4% of fALS patients and in less than 1% of sALS patients [61, 334] with an autosomal dominant inheritance pattern. The ubiquitously expressed DNA-/RNA-binding protein FUS localizes predominantly to the nucleus under physiological conditions [333]. FUS is involved in DNA repair [313] but also acts as an RNA-binding protein in several aspects of RNA metabolism including transcriptional regulation [188, 288], alternative splicing [121, 135, 164, 252], mRNA transport [96], mRNA stability [145, 297, 327], and microRNA biogenesis [105, 213]. Toxic GOF and LOF due to FUS aggregation and cytoplasmic mislocalization play a role in FUS-ALS/FTD pathogenesis [150]. An ASO-based therapeutic approach has been initiated for ALS caused by a specific *FUS* mutation (P525L) that is associated with an aggressive form of ALS with juvenile onset. Three of such FUS-ALS patients have received Jacifusen, a personalized ASO [13]. A FUS-ALS patient who already had developed respiratory problems received this personalized ASO treatment and died one year later [14]. The preliminary results from this case implicate, that adverse effects might also emerge by knocking down the wild type *FUS* transcript. FUS interacts as an RNA-binding protein with transcripts from about 5500 genes [164]. Thus, knocking down FUS via an ASO approach could interfere with the turnover of RNAs with long introns, many of which especially encode for synaptic proteins [164]. Downregulation of such transcripts and corresponding proteins in rodent primary neurons causes morphological alterations such as enlarged growth cones [225], shorter neurites [134, 225], abnormal dendritic spines [95, 327] and altered neurotransmission [297]. In vivo knockdown of FUS in murine hippocampal neurons causes increased phospho-tau accumulations as well as decreased neurogenesis, and thus a FTD-like phenotype [134, 297]. These data ultimately require enhanced efforts in the exploration of therapeutics which specifically target FUS expression. Jacifusen is scheduled to be given to eight additional patients with *FUS* mutations (Figueiredo, M. (2020)—Collaboration Funds Experimental Therapy for Rare FUS-ALS, accessed 3.28.20. https://alsnewstoday.com/2020/03/16/jacifusen-collaboration-funds-experimental-therapy-for-patients-with-rarefus-als/). Additional trials are planned with ION3763-CS1 which also targets this FUS mutation. Recruitment for this trial will start in June 2021, and there are so far no clinical data available. The relevant gene therapies for *SOD1*-, *C9ORF72*-, and *FUS*-ALS are depicted in Fig. 4.

#### Genetic modifiers in ALS

Ataxin-2 is a protein encoded by the *ATXN2* gene. Expansion of the polyglutamine tract in the human *ATXN2* gene leads to spinocerebellar ataxia type 2 (SCA2) [131, 238, 262]. SCA2 is characterized by neuronal degeneration in the cerebellum and inferior olive that causes ataxia, parkinsonism, and dementia. More than ten years ago, it has been discovered that this CAG repeat expansion is associated with a higher risk for ALS [76]. Since 2017, it is known that reduced levels of Ataxin-2 via ASO approaches extend life span and diminish functional and behavioral deficits in a TDP-43-ALS mouse model [21]. In September 2020, recruitment for a study of ALS patients with or without CAG expansion in the *ATXN2* gene has been started to assess safety, tolerability, and pharmacokinetics of the Ataxin-2 ASO termed BIIB105. The estimated study completion date is February 2023. The study is listed in https://clinicaltrials.gov.

### ASO treatments for muscular dystrophies

Duchenne's muscular dystrophy (DMD) and Becker's muscular dystrophy (BMD), the two forms of X-linked muscular dystrophy are caused by mutations in the Dystrophin gene. DMD occurs with an incidence of about 1:5000, whereas BMD affects children with an incidence of 1:30,000 [155]. The severe form, DMD, usually starts before 4 years. Affected boys lose ambulation around 12 years of age and get ventilation by the age of 18 [119]. The average life span today is 20–30 years due to advances in cardiac and respiratory care. In contrast to SMA, the spectrum of mutations in DMD is broad and ranges from point mutations to deletions as well as small insertions to large duplications [24].The dystrophin gene is one of the largest human genes containing 79 exons and approximately 2.4 million base pairs [2, 24, 41]. The primary role of the dystrophin protein is to link the actin-cytoskeleton with the extracellular matrix in cardiac and skeletal muscles by forming interactions with the subsarcolemmal actin and the large oligomeric dystrophin–glycoprotein complex (DPG). This regulates the proper functioning of muscle fibers. Defects of the DPG result in muscle weakness due to contraction-induced damage, necrosis, and inflammation, and a replacement of functional myofibers by fibrous and fatty connective tissue [29]. Disease severity depends very much on residual functions of the truncated dystrophin protein, which is derived from the mutant gene. Interestingly, mild forms of the disease have not only been detected in patients with point mutations which have only minor consequences for protein structure and function, but also in BMD patients in which multiple exon-encoded domains are not transcribed, resulting in a highly truncated dystrophin mRNA of only 8.8 kb [79]. This observation has paved the way to define essential subdomains within the dystrophin protein and exons within transcripts which are functionally important and needed to be present for mitigating disease severity. Similarly, these findings are the basis for the design of artificial mini-dystrophin genes which could be used for gene therapy through viral vectors which can only carry cDNAs of limited length [52].

#### Exon skipping

Retention or preservation of the open-reading frame (ORF) is an option to restrict the physiological consequences of dystrophin loss in DMD patients with nonsense mutations. Retention of the ORF can be mediated via ASO delivery. Binding of the ASOs to the dystrophin pre-mRNA transcript induces deletion/skipping of certain exon(s) and can thus restore the ORF [72]. The resulting shorter ORF produces a phenotype similar to that described for the milder form of DMD—the BMD. Three ASOs with phosphorodiamidate morpholino oligomer (PMO) backbone which are used for exon-skipping—eleplisen (exon 51), golodirsen (exon 53) and viltolarsen (exon 53)—have been approved by the FDA. However, the ASOs also show some limitations [75, 306] [1]. Since DMD patients have variants in many different exons that cause multiple reading frame disruptions, such single exon treatments are only applicable to a subset of DMD patients. Multi-exon skipping has been proposed to overcome the limited scope of single exon skipping by targeting DMD patients with variant exons [16]. A cocktail of ASOs targeting the variant hotspots of exons 45–55 can efficiently skip these exons in both immortalized DMD patient muscle cells and mouse models [73, 167]. However, a mixture of several ASOs also has a higher risk for off-target effects. This needs to be tested in clinical trials. If this finding is successfully translated to the clinic, it could potentially be useful for more than 65% of the DMD patients [74].

#### Genome editing

Genome editing with CRISPR-Cas9 appears as an attractive option for ORF restoration of the dystrophin gene. Application of CRISPR-Cas9 does not require re-injections because DNA instead of pre-mRNA is targeted. This approach could also be useful for treating patients with duplications in certain exons of the DMD gene, since it allows removal of extra exons and other gene insertions. The use of a multiplexed guide-RNA (gRNA) targeting the variant-prone exons 45–55 or 47–58, has been shown to restore dystrophin expression [306] in cultured patient-derived myoblasts. When these myoblasts were implanted into mice, expression was maintained [226]. However, because Cas9 induces double strand breaks (DSBs) by the gRNA in a targeted manner, off-target DNA-cutting remains an issue. Currently there are no clinical trials using genome editing approaches for DMD [75].

### Perspectives of AAV-based gene therapies beyond SMA

In the case of mutation-based LOF, relevant therapeutic approaches need to re-establish gene function. In the case of SMA, the scAAV9-*SMN1* gene therapy provided proof that this approach is feasible for treatment of a neurodegenerative disease. The dose of 1.1 × 10^14^ vg/kg body weight appeared sufficient to transduce the gene into a clinically relevant number of motoneurons, and to keep adverse effects such as severe acute liver injury at a low level. An increase of liver transaminase levels has been discussed as a consequence of a massive immune response against viral particles [195]. Unfortunately, in the case of another neuromuscular disorder—X-linked myotubular myopathy—the systemic delivery of a high dose of AAV8 particles containing the cDNA for myotubularin-1 was fatal. Two of six patients who received a dose of 2 × 10^14^ vg/kg or more died by progressive liver dysfunction followed by sepsis; it is presumed that AAVs directly damage liver cells [117]. Thus, AAVs are on one side effective gene-transfer-vehicles but on the other side bear the disadvantage of severe inflammatory reactions, particularly in systemic treatments with high doses. Strategies have been proposed to identify patients at risk for severe side effects [57, 58], and to overcome this problem by modulating the immune reaction towards a dampened response. This could be achieved either by depleting immunoglobulins via plasmapheresis; or even more specifically, by use of the IgG cleaving metalloproteases IdeS or IdeZ [63]. Such approaches to reduce AAV-autoantibodies could help to reduce side effects of AAV-based gene therapies for disorders such as DMD where systemic treatment with high numbers of virus particles is necessary. Furthermore, it can be beneficial for therapies of adult-onset disorders when patients are expected to have developed high AAV-antibody titers due to multiple previous exposures to such viruses.

Another strategy could be local injection, either by intrathecal application of the recombinant viruses or injection into the cisterna magna. This approach is currently followed with an AAV1-based gene therapy trial to increase expression of Progranulin in patients with frontotemporal dementia with granulin mutations [120] (Press release January 28th 2021: Passage Bio—Passage Bio Receives FDA Clearance of IND Application for PBFT02 Gene Therapy Candidate for Treatment of Patients with Frontotemporal Dementia with Granulin Mutations). It will show how the immune system reacts when AAV particles are injected into the CSF, how many brain cells can take up the viral particles to produce the transgene, and which levels of transgene expression are necessary for a clinically relevant effect.

In a study reported by Mueller et al., two ALS patients were treated with a single intrathecal infusion of AAVrh10 containing microRNAs to target *SOD1*. Downregulation of *SOD1* transcripts and protein was identified in spinal cord autopsy samples via Western blot in one of these patients. The same patient showed transient improvements in the strength of his right leg but no change in vital capacity, whereas the second patient maintained a stable vital capacity over the 12-month observation period [214]. The authors proposed that intrathecal infusion of AAV-delivered microRNAs for *SOD1* might have the potential for sustained beneficial effects, but possibly requiring immunsuppression.

### AAV-based micro-dystrophin (µDys) gene transfer

Causal therapies for DMD ideally should restore dystrophin expression in skeletal muscle. Unfortunately, the full-length dystrophin gene is too large for being packed into a functional AAV particle. Current strategies try to bypass this bottle neck by using truncated forms of dystrophin with residual function that is sufficient for attenuation of the disease [56]. Three independent clinical trials, using gene therapy with such micro-dystrophin constructs are currently ongoing (summarized in [52]. Initial results from one of these ongoing clinical trials with μDys showed that more than 80% of the muscle fibers were micro-dystrophin positive with significant expression of μDys in post-treatment biopsies (95.8% compared to normal) (Sarepta Therapeutics Announces Positive Updated Results from Micro-Dystrophin Trial to Treat Patients with DMD. https://investorrelations.sarepta.com/news-releases/news-release-details/sarepta-therapeutics-announces-23rd-internationalcongress-world), and summarized by [75, 306]. In addition, long-term therapy with μDys has beneficial effects in a canine model [165]. MicroDys does not contain all of the functional elements of the full-length dystrophin that are important for interactions with other proteins to convey forces on contractile actin. For this purpose novel variants of μDys with modified central rod domains, which are expected to exhibit higher cellular rescue activity, have been developed [240, 241] and are currently tested in clinical trials [178]. Another option which is currently followed is the use of miniaturised utrophin (μUtro), a shortened codon-optimized version of utrophin that differs in some protein–protein interactions from dystrophin. It prevents muscle pathology and appears in a non-immunogenic manner in large dog models. However, a positive effect on the disease phenotype has not been confirmed [280]. The overexpression of b1,4-N-acetylgalactosaminyltransferase (GALGT2) is a third possibility. GALGT2 stimulates the upregulation of key cytoskeletal binding proteins that can act as surrogates of dystrophin [43, 328]. After demonstrating safety in pre-clinical models, this therapy is now being tested in a phase I/IIa trial to evaluate its safety in humans. (Gene Transfer Clinical Trial to deliver rAAVrh74.MCK. GALGT2 for Duchenne Muscular Dystrophy-NCT03333590. https://clinicaltrials.gov/ct2/show/NCT03333590 48).

### AAV gene transfer in Parkinson disease (PD)

Parkinson's disease is a long-term degenerative disorder that affects multiple neuronal systems, in particular the motor system. The most common symptoms include tremor, rigidity, slowness of movement combined with walking difficulties. As the disease progresses, non-motor symptoms such as cognitive and behavioral alterations also become apparent. Motor symptoms are caused by degeneration of dopaminergic neurons in the substantia nigra. Loss of these dopaminergic neurons results in over-excitation of the subthalamic nucleus (STN) leading to increased inhibition of the thalamus [203]. Thus, Parkinson patients suffer from defects in movement initiation. Deep brain stimulation of the subthalamic nucleus appears as an attractive therapeutic option, because Levodopa [80] and other pharmacological treatments cannot halt the degenerative process in this disease. Hence, gene strategies using AAV vectors appear as an option for treatment of PD, also in combination with deep brain stimulation. Currently, there are several trials involving PD and gene therapy listed on clinicaltrials.gov. The all-encompassing approach of gene therapy for PD is to preserve the dopamine production in neuronal cells which are not affected, in order to functionally maintain the circuitry of the basal ganglia. For this purpose, direct intraparenchymal delivery via MRI-guided administration of AAV2 encoding the cDNA for aromatic L-amino acid decarboxylase (VY-AADC01) has been established [46]. Fifteen subjects with moderately advanced PD and drug refractory motor fluctuations received VY-AADC01. MRI-guided administration achieved putaminal coverage of 20–40% in accordance to increased enzyme activity assessed by PET and dose-related clinical improvements. Simultaneous reduction of antiparkinsonian medication led to reduced symptoms of dyskinesia [46]. A similar strategy is the MRI-guided AAV2-GAD (Glutamatdecarboxylase) delivery into the STN in an effort to increase local GABA inhibition and to correct pathological hyperactivity in this brain structure. Phase 1 and 2 trials have been performed with AAV2-GAD delivery via Convection-enhanced delivery (CED) to the STN of PD patients [146, 171]. These patients displayed improvements in their motor symptoms, but not to such an extent as could be achieved by deep brain stimulation of the STN [171].

### Gene therapy approaches in Alzheimer´s disease (AD)

Generation of the pathological amyloid-β (Aβ) peptide is believed to be the initial event in the AD process. Since several previous clinical trials including immune therapies to reduce the load with amyloid plaques failed or showed only minor effects [228], the FDA has now approved Aducanumab marketed under the name Aduhelm™ as an Aβ-directed antibody. The effects of this therapy appear to be less than expected, and this draws attention back to potential gene therapies which interfere with synaptic dysfunction and degeneration. Technical challenges for this approach are similar as in PD. Interventional MRI-guided convection-enhanced delivery is not exclusively relevant for treatment of PD patients. Likewise, AD patients could benefit from viral gene transfer and this technical approach. Some therapeutic developments focus on the tau protein [49, 329]. Pathological tau in AD brain is prevalent in a hyperphosphorylated state (i.e. phosphorylated at multiple sites within the tau protein). This posttranslational modification corresponds to tau aggregation and neurofibrillary tangle formation [133].

AAV-mediated gene transfer of the constitutively active tau kinase-p38γ has been shown to reduce tau-related dementia in pre-clinical dementia mouse models even when advanced cognitive deficits are present [136]. This strategy has not yet entered the stage of clinical trials for AD patients. However, it might become feasible since viral gene transfer of *BDNF* has entered the clinic. This trial is based on preclinical studies showing that BDNF delivery (via transgenic expression or infusions) in rodent models reverses synaptic loss after disease onset corresponding to improved learning and cognitive performance. BDNF also prevented lesion-induced entorhinal neuronal death in the primate model. In aged monkeys, BDNF improved performances in visuospatial discrimination tasks that correspond to increased mean entorhinal neuronal sizes [216]. Based on this data, a phase 1 clinical trial has started in February 2021. This trial assesses the effects of direct injection of AAV2-*BDNF* into the brain of AD patients or patients with Mild Cognitive Impairment (MCI) (Scott LaFee, First-in-Human clinical trial to assess gene therapy for Alzheimer’s Disease, UC San Diego News Center February 18th, 2021).

## Conclusions

Childhood proximal SMA is a genetically homogenous disease caused by lost or mutated *SMN1* and modulated by variable *SMN2* copies. This is an ideal condition to identify the underlying cellular defects and to develop therapeutic strategies compensating the lack of SMN protein, which is the key of SMA pathophysiology. Extensive research on this condition allowed the introduction of therapies with oligonucleotides and molecules that modulate pre-mRNA splicing. Additionally, AAV-based gene therapy entered the clinical stage for treatment of a neurodegenerative disease. Since most cases of SMA are diagnosed early in life and therapy usually starts immediately after diagnosis, immunological reactions against AAVs are less problematic compared to patients of advanced age being exposed to such viruses during their life. The clinical experience with these applications could help to develop and optimize analogous approaches beyond SMA. Currently, the development of therapeutic strategies is ongoing and hopefully will make a difference to the treatment of other neurodegenerative disorders such as amyotrophic lateral sclerosis, Parkinson’s and Alzheimer’s disease as well as muscular dystrophies.

## Data Availability

Not applicable.

## Abbreviations

3,4-DAP: 3,4-Diaminopyridine
4-AP: 4-Aminopyridine
AAV: Adeno associated virus
AD: Alzheimer's disease
ALS: Amyotrophic lateral sclerosis
ASO: Antisense oligonucleotide
ATXN2: Ataxin 2
ATP: Adenosine triphosphate
BBB: Blood-brain barrier
BDNF: Brain-derived neurotrophic factor
BMD: Becker muscular dystrophy
bp: Base pairs
CED: Convection-enhanced delivery
CHIP1: Calcineurin-like EF-hand Protein 1
CHOP INTEND: Children’s hospital of Philadelphia infant test of neuromuscular disorders
CMAP: Compound muscle action potential
CNTF: Ciliary neurotrophic factor
CSF: Cerebrospinal fluid
DMD: Duchenne muscular dystrophy
DPRs: Homopolymeric dipeptide repeat proteins
DSB: Double strand break
DSMA: Distal spinal muscular atrophy
E: Embryonic day
EAP: Expanded access program
EMA: European Medicines Agency
fALS: Familial amyotrophic lateral sclerosis
FDA: Food and drug administration
GABA: Gamma aminobutyric acid
GAD: Glutamate decarboxylase
GDNF: Glial cell-derived neurotrophic factor
GOF: Gain of function
gRNA: guide-RNA
HFMSE: Hammersmith functional motor scale expanded
hIGF-1: Complexed with rhIGFBP-3
HINE-2: Hammersmith infant neurological examination-2
IGF1: Insulin-like growth factor 1
iPSC: Induced pluripotent stem cell
ISS: Intronic splice silencer
JNKs: C-Jun N-terminal kinases
kb: Kilo base
LOF: Loss of function
LTFU: Long term follow up
MCI: Mild cognitive impairment
MMD: Microcystoid macular degeneration
MRI: Magnetic Resonance Imaging
NCALD: Neurocalcin delta
NMJ: Neuromuscular junction
OLE: Open-label extension
ORF: Open-reading frame
P: Postnatal day
PD: Parkinson's disease
PLS3: Plastin 3
PMO: Phosphorodiamidate morpholino oligomer
Ran: Repeat-associated non-AUG translation
RBP: RNA binding protein
RULM: Revised Upper Limb Module
sALS: Sporadic amyotrophic lateral sclerosis
scAAV: Self-complementary AAV
SMA: Spinal muscular atrophy
SMARD1: Spinal muscular atrophy with respiratory distress type 1
SMN: Survival Motor Neuron
SNP: Single nucleotide polymorphism
snRNP: Small nuclear ribonuclear particle
SOD1: Superoxid dismutase 1
STN: Subthalamic nucleus
TrkB: Tropomyosin-related kinase
